# Confirmatory Factor Analysis and Validity of the Sexual Harassment Scale in Football Refereeing

**DOI:** 10.3390/ijerph18041374

**Published:** 2021-02-03

**Authors:** Josefa Sánchez, Sara Serrat, Estefanía Castillo, Alberto Nuviala

**Affiliations:** 1 Department of Integrated Didactics, Area of Body Expression, School of Sport Education, Psychology and Sciences, University of Huelva, El Carmen Campus, Avenida de las Fuerzas Armadas s/n, 21007 Huelva, Spain; sara.serrat@alu.uhu.es (S.S.); estefania.castillo@dempc.uhu.es (E.C.); 2 Department of Sport Informatics, Area of Physical and Sport Education, School of Sport Sciences, Pablo de Olavide University, Ctra. De Utrera, km 1, 41013 Seville, Spain; anuvnuv@upo.es

**Keywords:** sexual harassment, mobbing, football referee, gender inequality, sport

## Abstract

Inequalities between men and women in the workplace are reflected in professional sports, specifically football refereeing. This phenomenon sometimes becomes sexual harassment since it is a stereotypically considered male profession in which women are a minority. To measure that behavior, it is necessary to count on valid and reliable tools. Therefore, the goal of this study was to determine the factorial structure and the discriminant and convergent validity of the ‘sexual experiences questionnaire’, version of the Department of Defence (SEQ-DoD). Eighty-nine male football referees and ninety-four female football referees, with a mean age of 23.30 ± 4.85 years, participated in this studio conducted questionnaire in Andalusia, Spain. A confirmatory factor analysis was performed using the robust maximum-likelihood estimation method. The goodness of fit was assessed, and the factorial invariance was calculated to determine the stability of the model. Subsequently, the validity was confirmed. The results corroborated the validity and reliability of the questionnaire adapted to the population studied. Therefore, it can be used as a research instrument.

## 1. Introduction

The MeToo Movement has contributed to raising awareness about the sexual harassment to which women are subjected. This harassment can occur in different environments, such as work, transportation, or public spaces, at home, at educational environments, or sports, among others [1,2,3,4,5].

Thousands of women have broken their silence and have shed light on sexual harassment in the workplace and public spaces [6]. Nonetheless, most incidents of sexual harassment seem to go unreported because of fear of retaliation [7], becoming an important social issue that prevents achieving gender equality in certain professions that have been considered to be masculine [8].

Sexual harassment is both an aggression against women and a form of gender discrimination [6]. Sexual harassment is defined as “any behavior, verbal or physical, of sexual nature that has the purpose or has the effect of undermining the dignity of a person, especially when an intimidating, degrading, or offensive environment is created” [9] (p. 12).

Sexual harassment can be divided into three dimensions that feature differences in their concepts, although they are related to each other [10]. These dimensions are sexual coercion; unwanted sexual attention, and gender-based harassment. Sexual coercion is sexual cooperation in exchange for certain considerations, making itself visible through bribes, threats, and sexual blackmail. Unwanted sexual attention refers to verbal and non-verbal unwanted, offensive behaviors, without being reciprocal, such as invitations to dates despite saying no, inappropriate touching, or non-consensual sex. Gender-based harassment refers to verbal and non-verbal behaviors with the purpose of insulting, harassing, and degrading through disrespect, sexist comments, distinctive treatment, comments about the body, debate about their sexual life, gestures, and inappropriate exhibitions [11].

Sexual harassment victims suffer a violation of their dignity. They lose the right to enjoy their jobs and may have negative consequences on their physical and mental health. In addition, their performance and productivity may decrease [12,13,14].

Different forms of sexual harassment occur in all sports and at all levels [15], and although prevalence rates have not been systematically estimated, it appears to be higher on the elite level of sports [16]. Traditionally, the figure of the bully has been the coach, but today it is also considered that there is sexual abuse between equals and that it is independent of their sexual orientation [16]. In the world of sports, there have been numerous studies on violence in soccer [17,18,19,20,21,22]. Although few studies address sexual violence, they are not always focused on the female population [23,24]. These studies often focus on the female population as victims of domestic violence after soccer games, but not as professional athletes [25,26].

In relation to previous studies on sexual harassment, research has been carried out on sports in general [27,28] or on university sports [7]. On the other hand, studies on individual sports, such as athletics [29,30,31], in sports teams, such as American football [23], or soccer [32]. These studies show, among other findings, that this type of behavior on athletes can cause serious consequences on the physical and mental health of the victims [16,29,33].

In relation to soccer, studies on refereeing reveal the stress to which the referees are exposed in their professional work, and on occasions, the abuse they suffer from players, coaches, and spectators [34,35,36,37]. Although some studies have been carried out in relation to the refereeing figure in soccer, research lacks in this regard [38]. Refereeing work is one of the least valued professions in the soccer world [39,40,41], and it is a highly masculinized context [42,43], with the number of women being considerably lower than men [44,45]. On the other hand, studies show that female referees suffer greater pressure during matches and less recognition of their work, thus hindering their professional development and suffering double discrimination for being a referee and for being a woman [44,46,47].

To study sexual harassment in sports, qualitative instruments, such as interviews or life history, have been used [23,28,30,31,46,48]. Timpka et al. [31], designed a protocol for the prevention of sexual abuse in sports. After reviewing the literature, the SEQ-DoD questionnaire was considered more accessible and easy to translate and, therefore, to apply to this study. This questionnaire has been widely used. It has also been subjected to different reviews and critics that conclude that it is an adequate instrument due to the scarcity of quantitative measures that exist to this topic of sexual harassment [49].

Another of the main reasons to use this questionnaire is the similarity between the sports and the military fields. One of the characteristics of these work environments is the presence of women and men in a context historically understood for men [46].

The military and sports worlds also coincide in the minimum presence of women and the traditional social thought that both are reserved for male practice only [44,50,51].

This questionnaire has been widely used in different countries and populations, especially those related to the military field, but it has also been applied in other populations [10,52].

In the absence of a sexual harassment investigation in the football refereeing world, the purpose of the present study was to adapt the ‘sexual experiences questionnaire’ version of the Department of Defense (SEQ-DoD) [11]. The goals were to determine the factorial structure and the discriminant and convergent validity of the SEQ-DoD. 

The most relevant contribution of this paper is to adapt an instrument to collect useful information in the sports context, specifically in football refereeing. It may be used in future studies, adapting it to other sports or other figures, such as female and male players, or technical team, among other future lines of research.

## 2. Materials and Methods

### 2.1. Participants

One hundred and eighty-three183 football referees from different categories participated in the present study, of which 51.4% were women. The mean age was 23.30 ± 4.85 years, and the average experience in refereeing was 5.32 ± 4.80 years.

### 2.2. Instrument

The SEQ-DoD (the ‘sexual experiences questionnaire’ version of the Department of Defense) [11] was the instrument used in the present study. It is a measurement tool used to determine offensive sexual experiences. This questionnaire was reviewed and adapted to the football refereeing environment. The SEQ-DoD, in its original version, consisted of four factors, namely: sexist hostility, sexual hostility, unwanted sexual attention, and sexual coercion. All the questions shared a common root: “In the last 12 months, have you observed or been a victim of some type of behavior described below, perpetrated by others in your work as a football referee?” The body of each element described behaviors that the interviewee might have experienced. The reliability of the instrument, after fieldwork, measured with Cronbach’s alpha, was 0.934. Responses were given on a Likert-type scale, ranging from 1 (never) to 5 (very often). Various sociodemographic questions were added to the questionnaire, such as sex, age, experience in football refereeing, and refereeing category.

### 2.3. Procedure

First, the organization responsible for the football refereeing that participated in the study was informed about it. Participating referees were asked for permission to request their informed consent. The study was conducted after approval. The design took into account the principles established in the Declaration of Helsinki [53]. In the same way, we took into consideration the current Spanish legal regulations that normalize the protection of personal data [54]. The fieldwork was carried out by means of a self-administered questionnaire with the presence of an interviewer, which lasted about ten minutes.

### 2.4. Statistical Analysis

First, we performed a confirmatory factor analysis. The method used was the robust maximum-likelihood estimation. To determine the goodness of fit, we reviewed the indicators, namely: the Chi-square value divided by the degrees of freedom (χ^2^/gl), values below 5.00 were considered acceptable; root mean square error of approximation (RMSEA), the model would show an acceptable adjustment if the value were <0.07; and comparative fit index (CFI), values above 0.90 are considered acceptable [55,56]. In addition, in order to follow Byrne’s indications [57], we added the Akaike information criterion and the expected cross-validation index. Subsequently, the factorial invariance was calculated to determine the stability of the model in different populations.

Convergent validity tests were performed by calculating correlations between factors and composite reliability. Finally, we determined the discriminant validity using three different procedures: calculation of correlations between factors and comparison with the square root of the average variance extracted (AVE); estimation of alternative models; and construction of confidence intervals for factors correlation with 95% confidence interval. The statistical analyses were performed using the statistical packages SPSS (Statistical Package for the Social Sciences, version 23.0 (SPSS, IBM, Armonk, NY, USA)) and AMOS v23 (Analysis of Moment Structure (AMOS, IBM, Armonk, NY, USA), version 23.0)

## 3. Results

**Figure 1:**
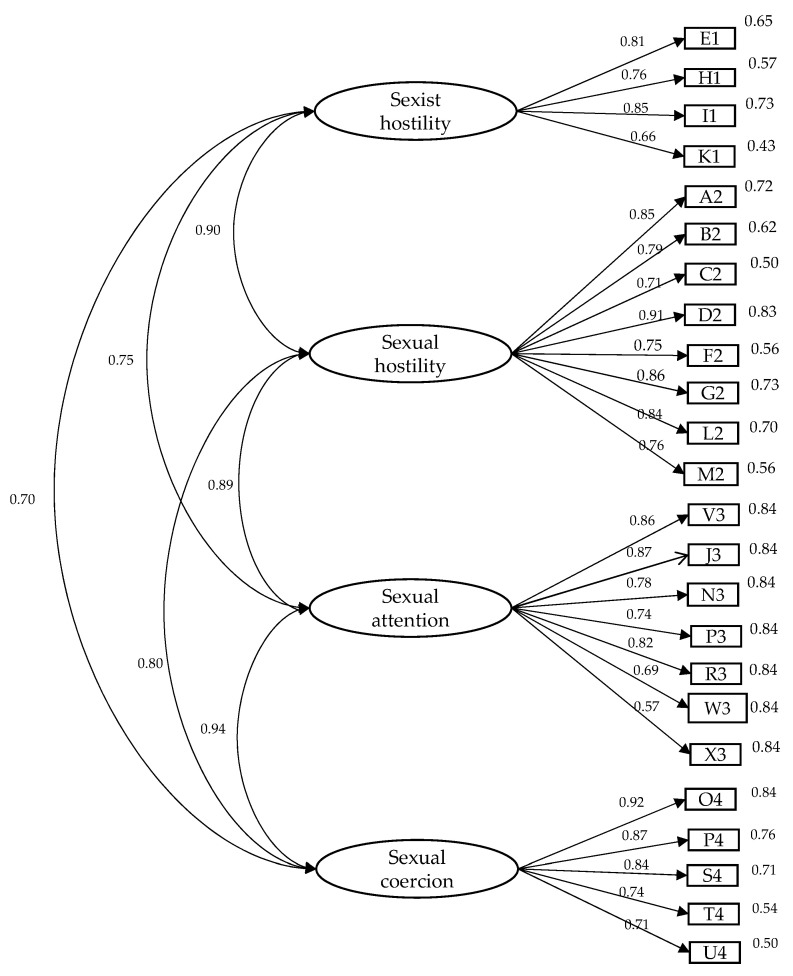
Structural model of ‘sexual experiences questionnaire’, Department of Defence (SEQ-DoD).

To confirm whether or not the scale met the expected factorial structure, we performed a confirmatory factor analysis (Figure 1). The adequacy of the model under test (model 0), which consisted of four factors and twenty-four items, was carried out through a joint assessment of a group of indices. Table 1 contains the information provided by the adjustment indices, and it can be concluded that it was a correct model.

The factorial invariance of the model was contrasted by comparing two groups of football referees, which were selected at random among the population object of the present study. We considered the differences in χ^2^ between the models without restrictions (Model 1), Model 2 (had restrictions relating to the weight measurement), and Model 3 (had weight measurement and covariance restricted), observing differences between models 1 vs. 2 and 2 vs. 3 (Table 1) The CFI value of the models indicated that all had very similar values, with a difference between them equal to −0.01. Similarly, the Akaike information criterion and the expected cross-validation index indicated that the differences in the adjustments were minimal; therefore, the different models exhibited very similar values. These results suggest the factorial invariance of the model.

The convergent validity was confirmed by the calculation of the correlations between the factors of the SEQ-DoD. The results indicated positive and significant correlations between the factors of the scale. Similarly, the composite reliability values obtained for each dimension suggested the existence of this type of validity.To determine the discriminant validity, the square root of the AVE was compared with the correlation between both constructs. Table 2 shows this correlation and, in the diagonal, the square root of the AVE, which was superior to the correlation between the different constructs of the questionnaire. Considering these results, it can be affirmed that there was discriminant validity.

As a second discriminant validity test, alternative models were estimated in such a way that a restriction in all of them, i.e., the correlation between each pair of dimensions, should be equal to 1. In addition, the chi-square test was performed with each one to compare the models to assess whether or not they were significantly different. Table 3 shows how the difference between the chi-square test values was always significant. This way, the dimensions of the scale were significantly different from each other, thus confirming the discriminant validity.

As a third way to confirm this type of validity, we calculated the possible correlations between the factors. This procedure allowed the construction of the confidence interval relating to the correlations between the dimensions. Table 3 shows that the discriminant validity of the scale could be confirmed, since none of the confidence intervals of these correlations contained value 1 at 95% confidence.

## 4. Discussion

The goals of the present study were to determine the factorial structure and the discriminant and convergent validity of the SEQ-DoD [11] in Spanish football referees. The results confirmed the validity and reliability of the adaptation of the questionnaire to the population under study. The resulting latent variables were the same as those in the original questionnaire.

The purpose of our study was to determine the fit of the original model to the data obtained from a sample of Spanish football referees. To that end, we performed a confirmatory factor analysis. The parameters were estimated using the maximum likelihood method [58]. To assess the adequacy of the model under test, we performed a joint assessment of a group of indices. Some of the most used adjustment indices were selected considering values above 0.90 acceptable in the case of the CFI. In the case of RMSEA, the model would exhibit an acceptable fit if the value was <0.07 [59], and values ≤ 0.06 would indicate a good fit [60]. Regarding the values of the quotient between χ^2^ and gL, in a model considered perfect, the value would be 1.00, and ratios below 2.00 would be considered a very good fit of the model, whereas values below 5.00 would be considered acceptable [60,61,62]. Finally, due to the convenience of comparing the fit of the model, we added two specially developed indices, namely: the Akaike information criterion, i.e., a comparative index between models, having to choose the model that presents a lower value [63] (values closer to zero indicate a better fit); and the expected cross-validation index, which measures the discrepancy between the covariance matrix involved in the analyzed sample and the expected covariance matrix of another sample of the same size. When models are being compared, a lower expected cross-validation index value indicates the model with the best fit [63].

The results of the different fit indices of the original model can be considered acceptable. Therefore, the model can be considered correct for the population of football referees assessed in the present study. Furthermore, the reliability of the resulting instrument measured with Cronbach’s alpha was 0.934, which indicated good internal consistency.

Subsequently, we assessed the invariance of the factorial structure through multi-group analysis [64]. To that end, the group was divided into two subgroups at random. The aim was to confirm that there were no significant differences between a model without invariance and different models with invariance in some parameters. We found significant differences in chi-square values between the unrestricted model (Model 1) and the rest of the models. However, given that the chi-square coefficient is sensitive to sample size, we also used the criterion proposed by Cheung and Rensvold [65] with respect to ΔCFI. According to these authors, ΔCFI values lower than or equal to −0.01 indicate that the null hypothesis of invariance cannot be rejected. The ΔCFI values found in the present study, in the comparison of the unrestricted model with the rest of the models, suggest that the factorial structure of the scale was invariant.

The convergent validity was determined by the correlations between the SEQ-DoD factors using Pearson’s correlation coefficient. The correlations between them were positive, being high in some cases, which can give an idea of the similarity of the constructs. The results of the correlations demonstrated this type of validity since the results were within the criteria proposed by Devon et al. [66] for this type of validity. The second test of convergent validity of the instrument was determined by composite reliability. Acceptable values are >0.6 [67,68]. Both tests indicated the existence of this type of validity.

The discriminant validity of the scale was expressed by the contrast between the different factors that composed it. This type of validity occurs if the concepts that comprise it are really different and, at the same time, related to each other [69]. To confirm this validity, these concepts were assessed in various ways. The first consisted of comparing the square root of the AVE with the correlation between the constructs of the scale [67]. The square root of the AVE should be higher than the correlation between the constructs so that there is discriminant validity between them. Considering the results of the correlations and the AVE values, it can be affirmed that there was discriminant validity.

This type of validity can also be confirmed in two other ways. The first has been proposed by Burnkrant and Page [70]. It attempts to estimate alternative models in such a way that a restriction is included in all of them, i.e., the correlation between each pair of dimensions should be equal to 1. In the other, each model should be subject to a chi-square test to compare them and assess whether they are significantly different. Our results have proven that the difference between the chi-squared values was always significant. Therefore, the dimensions of the SEQ-DoD were different from each other, thus confirming the discriminant validity.

The third way consists of calculating the possible correlations between the factors and constructing the confidence intervals of the correlations between all the dimensions. The results of the present study also indicated the occurrence of this type of validity since none of the confidence intervals of these correlations contained the value 1 at 95% confidence [71].

## 5. Conclusions

In conclusion, the SEQ-DoD has proven to be valid and reliable. However, it is still in an early stage. The limitations related to the number of football referees assessed in the fieldwork and the lack of bibliography on the subject makes it necessary to conduct further studies in-depth and improve, if possible, this instrument.

## Figures and Tables

**Table 1:** Statistics adjustment for the ‘sexual experiences questionnaire’, Department of Defence (SEQ-DoD) scale model; comparison between models using Model 1 as correct.

Goodness-of-Fit Indices and Model Comparisons of the Tested Models											
Model	CMIN	DF	*p*		CMIN/DF	CFI		RMSEA	ECVI		AIC
Model 0	332.997	238	<0.001		1.399	0.930		0.063	4.570		456.997
Model 1	596.642	476	<0.001		1.253	0.913		0.051	8.532		844.642
Model 2	630.864	496	<0.001		1.272	0.903		0.052	8.473		838.864
Model 3	640.836	506	<0.001		1.266	0.903		0.052	8.372		828.836
**Comparisons of Conditions Using Measurement Invariance Procedures**											
	**Model**			**Dif. DF**			**Dif. CMIN**			***p***	
Assuming that model 1 is correct	2			20			34.222			0.025	
	3			30			44.194			0.046	
Assuming that model 2 is correct	3			10			9.972			0.443	

Note. CMIN: minimum discrepancy; DF: degrees of freedom; CFI: comparative fit index; RMSEA: root mean square error of approximation; ECVI: expected cross-validation index; AIC: Akaike information criterion; Model 1 had no restrictions; Model 2 had restrictions relating to the weight measurement; Model 3 had weight measurement and covariance restricted; Dif. CMIN: difference between model 1 and the rest of the models; Dif. DF: difference between model 1 and the rest of the models; *p*: significance level between models.

**Table 2:** Means, correlations between factors, and square roots of average variance extracted (In the diagonal); Cronbach’s Alpha; composite reliability.

	Total Mean	Sexist Hostility	Sexual Hostility	Unwanted Sexual Attention	Sexual Coercion	α	CR
Sexist hostility	2.46 ± 1.04	(0.716)	0.612 **	0.485 **	0.427 **	0.803	0.806
Sexual hostility	2.23 ± 0.95		(0.735)	0.602 **	0.525 **	0.899	0.901
Unwanted sexual attention	1.44 ± 0.60			(0.629)	0.599 **	0.825	0.817
Sexual coercion	1.22 ± 0.54				(0.714)	0.830	0.837

*Note.* ** *p* < 0.01; CR: composite reliability.

**Table 3:** Test of χ^2^ differences; confidence intervals of correlations between dimensions.

	χ^2^ Differences (gL)	*p*	Confidence Interval
Sexist Hostility/Sexual Hostility	334.129 (239) − 332.997 (238) = 1.132 (1)	<0.001	(0.679–0.815)
Sexist Hostility/Unwanted Sexual Attention	351.000 (239) − 332.997 (238) = 18.003 (1)	<0.001	(0.351–0.600)
Sexist Hostility/Sexual Coercion	360.712 (239) − 332.997 (238) = 27.715 (1)	<0.001	(0.286–0.553)
Sexual Hostility/Unwanted Sexual Attention	344.250 (239) − 332.997 (238) = 11.253 (1)	<0.001	(0.577–0.750)
Sexual Hostility/Sexual Coercion	355.737 (239) − 332.997 (238) = 22.74 (1)	<0.001	(0.400–0.631)
Unwanted Sexual Attention/Sexual Coercion	367.236 (239) − 332.997 (238) = 34.239 (1)	<0.001	(0.503–0.828)

## Data Availability

Data is contained within the article; there is no supplementary material.
